# Molecular consequences of fetal alcohol exposure on amniotic exosomal miRNAs with functional implications for stem cell potency and differentiation

**DOI:** 10.1371/journal.pone.0242276

**Published:** 2020-11-16

**Authors:** Honey Tavanasefat, Feng Li, Kikuye Koyano, Bahar Khalilian Gourtani, Vincent Marty, Yatendra Mulpuri, Sung Hee Lee, Ki-Hyuk Shin, David T. W. Wong, Xinshu Xiao, Igor Spigelman, Yong Kim

**Affiliations:** 1 Laboratory of Stem Cell & Cancer Epigenetic Research, School of Dentistry, UCLA, Los Angeles, California, United States of America; 2 CSUN-UCLA Stem Cell Research Bridge Program, Department of Biology, California State University at Northridge, Northridge, California, United States of America; 3 Division of Oral Biology & Medicine, UCLA School of Dentistry, Los Angeles, California, United States of America; 4 Department of Integrative Biology and Physiology, Molecular Biology Institute, University of California Los Angeles, Los Angeles, California, United States of America; 5 The Shapiro Family Laboratory of Viral Oncology and Aging Research, UCLA School of Dentistry, Los Angeles, California, United States of America; 6 UCLA Broad Stem Cell Research Center, Los Angeles, California, United States of America; Università degli Studi della Campania, ITALY

## Abstract

Alcohol (ethanol, EtOH) consumption during pregnancy can result in fetal alcohol spectrum disorders (FASDs), which are characterized by prenatal and postnatal growth restriction and craniofacial dysmorphology. Recently, cell-derived extracellular vesicles, including exosomes and microvesicles containing several species of RNAs (exRNAs), have emerged as a mechanism of cell-to-cell communication. However, EtOH’s effects on the biogenesis and function of non-coding exRNAs during fetal development have not been explored. Therefore, we studied the effects of maternal EtOH exposure on the composition of exosomal RNAs in the amniotic fluid (AF) using rat fetal alcohol exposure (FAE) model. Through RNA-Seq analysis we identified and verified AF exosomal miRNAs with differential expression levels specifically associated with maternal EtOH exposure. Uptake of purified FAE AF exosomes by rBMSCs resulted in significant alteration of molecular markers associated with osteogenic differentiation of rBMSCs. We also determined putative functional roles for AF exosomal miRNAs (miR-199a-3p, miR-214-3p and let-7g) that are dysregulated by FAE in osteogenic differentiation of rBMSCs. Our results demonstrate that FAE alters AF exosomal miRNAs and that exosomal transfer of dysregulated miRNAs has significant molecular effects on stem cell regulation and differentiation. Our results further suggest the usefulness of assessing molecular alterations in AF exRNAs to study the mechanisms of FAE teratogenesis that should be further investigated by using an *in vivo* model.

## Introduction

Alcohol (ethanol, EtOH) consumption is recognized as the leading preventable cause of birth defects and intellectual diability [1]. High levels of alcohol consumption during pregnancy can result in fetal alcohol spectrum disorders (FASDs), which are characterized by prenatal and postnatal growth restriction, craniofacial dysmorphology and structural and functional abnormalities of the central nervous system [2]. While the developmental defects from alcohol abuse during gestation have been described, the specific mechanisms by which alcohol mediates these injuries have yet to be determined [3, 4]. This is an important question to address if we are to identify affected children at an early age and intervene to prevent or mitigate the damage. Better understanding of the pathophysiological mechanisms of EtOH-induced teratogenesis should be useful in the development of biomarkers for early detection and interventions to prevent or mitigate EtOH’s teratogenic effects.

The importance of the orchestrated interplay between molecular regulators in stem cells has been demonstrated in the maintenance of self-renewing pluripotency or the initiation of differentiation. In addition to stem cell intrinsic factors, cellular communications with the stem cell niche have been demonstrated to play an important role in stem cell maintenance and development. Accumulating data have demonstrated that the beneficial effects of stem cells are not restricted to their ability to differentiate into mature tissues but are also due to their ability to release a multitude of molecules. Stem cells may release potent combinations of factors that modulate the molecular composition of the cellular environment and evoke a multitude of responses from neighboring cells. Stem cells are especially vulnerable to EtOH toxicity through decreases in pluripotency, survival capacity, and/or altered differentiation [5]. We recently described the molecular signatures of EtOH’s effects on pluripotency and differentiation of human embryonic stem cells (hESCs), hESC-derived neuronal stem cells, as well as adult dental pulp mesenchymal stem cells (MSCs) [6–8]. Studies also showed that EtOH exposure alters the differentiation potential of amniotic fluid-derived stem cells [9] and reduces neuronal stem cell numbers in developing and adult brains [10, 11].

Recently, cell-derived extracellular vesicles, including exosomes and microvesicles, have emerged as a mechanism of cell-to-cell communication [12, 13]. Extracellular vesicles can contain several species of RNAs (exRNAs), including mRNA, microRNA (miRNA) and long non-coding RNA [14]. Exosomes are found in various body fluids including serum, urine, saliva, and amniotic fluid [14–18]. Secreted exRNAs have been demonstrated to act as paracrine/endocrine mediators, capable of modifying the phenotype of recipient cells with functional consequences. Furthermore, regenerative roles of the stem cell-derived exosomes have been demonstrated [19–22]. Thus, it is reasonable to hypothesize that the physiological status of pregnancy and any pathological changes that might occur during pregnancy, such as those due to maternal alcohol exposure, should be reflected in the exRNA profiles of maternal biofluids, and may even be partially mediated by exRNA signals. Research on the mechanisms of exRNA signaling in pregnancy may unveil the potential of exRNAs as biomarkers for prediction and diagnosis of adverse pregnancy outcomes.

The effect of EtOH on exRNAs has not been extensively studied, but accumulating evidence supports the likelihood of EtOH’s effects on the biogenesis and functions of exosomes and associated exRNAs. It was shown that circulating miRNAs (localized in exosomes) are increased in alcoholic liver disease [23]. Also, EtOH greatly increased the production of exosomes and had a significant effect on protein content in cardiomyocyte-derived exosomes [24]. EtOH exposure significantly elevates levels of a subset of miRNAs in secreted extracellular vesicles in fetal neural stem cells [25]. Studies have shown that alcohol use during pregnancy resulted in alterations in serum miRNA in pregnant women [26, 27]. However, it is currently not known if EtOH has any effect on the biogenesis and bio-function of non-coding exRNA during fetal development.

Amniotic fluid (AF) is initially made by the mother until the fetal kidneys start contributing to the AF by urinating into it. The fetus also ingests AF, which passes through the digestive system and into the kidneys, and back out again to the AF sac as urine. AF is a rich source of fetal cell-free DNA and RNA [28, 29]. Composition of nucleic acids in AF has been demonstrated to be distinct from those in maternal blood [29]. It primarily contains cell-free nucleic acids from the fetus itself while circulating cell-free fetal nucleic acids are of trophoblast origin [29–31]. They are relatively free of maternal nucleic acids, as maternal-fetal nucleic acid trafficking is overwhelmingly unidirectional from fetus to mother [32].

Exosomal exRNA from human AF has been purified and characterized [16–18]. Human AF contains about 30 ng/μl of RNA which contains a large number of detectable miRNA species (359 miRNAs), but the composition of these miRNAs did not show substantial overlap with the other maternal body fluids tested [33]. This may result from a filtering process by the placenta that reduces the content exchange between amniotic fluid and other body fluids. Another study showed that amniotic exRNA can be used to determine fetal sex [17]. These findings qualify AF as a rich source of exRNA to monitor the physiological status of the fetus.

In our previous studies, we utilized an *in vivo* model of fetal alcohol exposure (FAE) to address the behavioral and neurophysiological consequences of FAE [34] and an *in vitro* model where we performed a genome-wide analysis of EtOH’s effects on the maintenance and differentiation of human embryonic stem cells (hESCs) in culture [35]. The goals of the current study were to define the effect of FAE on the biogenesis of amniotic exRNAs and how this might affect stem cell maintenance and development. Defining the effect of FAE on amniotic exRNA content may help with the development of biomarkers for early detection and interventions to prevent or mitigate EtOH’s teratogenic effects.

## Materials and methods

### EtOH treatment of pregnant rats and collection of amniotic fluid

To determine the effect of EtOH on the content of exRNA in the amniotic fluid, we used our model of defined EtOH exposure. All experiments using animals were approved and performed in accordance with the UCLA Institutional Animal Care and Use Committee, known as the Chancellor’s Animal Research Committee (ARC) (ARC # 2008-131-21C). Animals received daily consideration for their bodily comfort. They were treated kindly, properly fed and their surroundings maintained in the best possible sanitary condition. This study had no pain/distress to the mice since euthanasia was employed for harvesting of tissues only without recovery surgery. We did not observe any mortality related to our experimental procedure. Euthanasia on mice was carried out according to the current AVMA guidelines for the Euthanasia of Animals.

In this model, timed pregnant Sprague Dawley rats (Envigo, Placentia, CA) were administered 20% w/v of EtOH in water (0 or 2.5 g/kg, oral gavage) on days 5, 8, 10, 12 and 15 of pregnancy [34]. We collected amniotic fluid from pregnant Sprague Dawley dams as described with modifications [36, 37]. The level of AF in rats is known to increase at day 10 and decrease after day 18 [38]. Pregnant dams at E13, E16 or E19 of gestation were anesthetized with isoflurane and euthanized by decapitation. Thus, the duration of FAE was 8, 11 or 14 days. The uterus was exposed, and individual fetuses dissected from the uterus with embryonic membranes intact. AF was collected by insertion of a 29^1/2^-gauge needle directly into the amniotic cavity, carefully avoiding puncture of vitelline or allantoic vessels. Pooled AF from all embryos (both male and female fetuses) from the same dam were stored at -20 °C. Individual embryos were freed from the embryonic membranes and stored at -20 °C.

### Exosome isolation and characterization

Exosomes were isolated by differential centrifugation as previously described [39]. AFs (0.5–1.0 ml) were spun for 20 min at 300 × g to remove contaminating cells. The supernatant was collected and centrifuged at 10,000 × g for 30 min to remove cellular debris and microvesicles. Exosomes were pelleted using a Beckmann ultracentrifuge at 120,000 × g for >18 hrs as described in our previous work [40]. The pellets were washed with PBS, pooled, and ultra-centrifuged at 120,000 × g for >4 hrs. The final pellet was re-suspended in PBS. Purification of exosomes was confirmed by electron microscopy and in addition, enrichment of exosomes was determined by Western blot analysis of CD24, a known marker for exosomes in urine and amniotic fluid [41], as well as CD9 and CD63 [42] content. The quantity of isolated exosomes was determined by using ExoELISA kit for CD63 (SBI, Palo Alto, CA).

### Western blot

After purification of protein samples according to the standard protocol, the concentration of protein was determined BCA assay (Bio-Rad, Hercules, CA). SDS-PAGE gel electrophoresis and blotting was performed and membranes were blocked in 3% milk in TBST and probed with the following antibodies: anti-CD9 (1:3,000, #C12162, Assay Biotech, Fremont, CA), anti-CD24 (1:3,000, sc-33669, Santa Cruz Biotech, Santa Cruz, CA), anti-CD63 (1:3,000, MX-49, sc-5275, Santa Cruz Biotech, Santa Cruz, CA), anti-Calnexin (1:3,000, #ab22595, Abcam, Burlingame, CA), and anti-β-actin (1:5,000, Sigma, St. Louis, MO) for overnight in 4°C. Membranes were then washed with TBST followed by incubation with horseradish peroxidase-conjugated secondary antibody (1:3,000, #NA931V and #NA934V, GE Healthcare, Pittsburgh, PA) for 1 h at room temperature. Membranes were developed with an enhanced chemiluminescence (ECL) reagent (Amersham Biosciences, Little Chalfont, UK) and HyBlot CL films (Denville Scientific Inc., Holliston, MA).

### RNA isolation and next generation sequencing

AF exosomal RNA was isolated by using miRNeasy micro kit (Qiagen, Germantown, MD) according to the manufacturer’s instructions. We have used the protocol for miRNA-enriched fractions of <200 nt. The quality and quantity of RNA was determined by Bioanalyzer analysis (Agilent, Santa Clara, CA). Small RNA library was constructed by using NEBNext Small RNA Library Prep set for Illumina (New England Biolabs, Ipswich, MA). Briefly, exosomal RNA (20 ng each) was mixed with 1/15 amount of Spike-in RNA (Exiqon-Qiagen, Germantown, MD) and used for library construction. The quality of amplified library was checked by Bioanalyzer analysis and size selection was performed by gel electrophoresis. Again, the quality of purified library was checked by Bioanalyzer and NGS was performed with Illumina HiSeq 3000 (single read, 10 million reads in the depth of coverage and 1X50 read length). The resulting RNA-Seq data has been deposited to Gene Expression Omnibus (GSE132437).

### Bioinformatics analysis

Adapters and low-quality nucleotides were removed from raw fastq sequences using Cutadapt (v.1.11). Adapter-free sequences were mapped to the Rat genome (rn5) using bowtie [43] (v.1.1.2) using the parameters ‘-v 1 –k 100 –best–strata’. Mapped reads were annotated to mature rat miRNA and piRNA. Rat miRNA annotations (rn5) were downloaded from miRBase [44] (v.21). Rat piRNA annotations (rn4) were downloaded from piRNABank, v.1 [45]. Liftover [46] of piRNA annotations from rn4 to rn5 was performed since rn5 annotations were not available. Bowtie alignments were assigned to annotated small RNA species by requiring the alignment start site to be between -3nt to +2nt positions near the annotated start site and the alignment end site be between -2nt and +3nt of the annotated end site. Multi-mapped reads are distributed 1/*n*, where *n* is the number of mapping locations. DESeq2 [47] (version 1.14.1) was used to further normalize and determine differential expression of miRNA and piRNA species between control and EtOH-treated groups for Day 16 and Day 19. Reads were aligned to the spike-in control sequences, allowing no mismatches. The number of reads mapped to each miRNA was normalized with the spike-in controls and the total number of mapped reads in each library. Spike-in normalized miRNA species counts from each sample were normalized again using DESeq2’s scale factor. The piRNAs were normalized with the miRNA species’ counts and miRNAs were removed for differential expression analysis. Species found to be differentially expressed between the control and EtOH-treated groups (*p*-value < 0.05) were chosen for further experimental validation.

### qRT-PCR verification

Quantitative PCR analysis was done by preparing cDNA with QuantiMir kit (SBI, Palo Alto, CA). This kit utilizes highly efficient poly-A tailing and reverse transcription in a single reaction tube that provides uniform cDNA synthesis of miRNAs. The first-strand cDNAs with anchor-tailed miRNAs were subjected to PCR analysis with specific mature miRNA primers (primer sequences shown in S1 Table in S1 File) and the universal reverse primer (SBI, Palo Alto, CA). qPCR was performed in 10 μl reaction containing miRNA primer: the universal primer (2:1) in Roche 480. We used RNU1A housekeeping small RNA as a reference control assay.

### Culture of rat bone marrow stem cells and EtOH treatment

Primary culture (P2) of Sprague Dawley rat bone marrow mesenchymal stem cells were purchased from Cyagen (Cat# RASMX-01001) (Santa Clara, CA) and cultured in α-minimal essential medium (MEM) supplemented with 10% (v/v) fetal bovine serum (FBS), 100 μM L-ascorbate-2-phosphate, 2 mM L-glutamine, 50 U/ml penicillin, 50 μg/ml streptomycin, and 50 μM β-mercaptoethanol. Cells were expanded and cells under P5 were used for experiments. Osteogenic induction and EtOH treatment were done as described in our previous work [48]. For EtOH treatment, cells were cultured in the complete culture medium containing EtOH diluted to a desired concentration for an indicated time period with a daily medium change.

### *In vitro* osteogenic differentiation

Cells were cultured in osteogenic medium containing 10 mM β-glycerophosphate (Sigma-Aldrich), 100 μM L-ascorbic acid 2-phosphate (Wako), and 100 nM dexamethasone (Sigma) for indicated time periods. For alkaline phosphatase staining, after 4 weeks of induction, cells were fixed with 4% paraformaldehyde for 1 min at room temperature and incubated with a solution of 0.25% naphthol AS-BI phosphate and 0.75% Fast Blue BB (Sigma-Aldrich, St Louis, MO, USA) dissolved in 0.1 M Tris buffer (pH 9.3). For Alizarin Red staining to detect mineralized nodule formation, cells were fixed cells for 30 min at room temperature and incubated with 2% Alizarin Red (Sigma-Aldrich, St Louis, MO, USA).

### Exosome labeling and uptake

Exosomes were labeled with PKH26 (Sigma Aldrich, St. Louis, MO), according to the manufacturer’s protocol, with some modifications. Briefly, exosome pellets were re-suspended in 1 ml Diluent C. Separately, 1 ml Diluent C was mixed with 4 μl PKH26. The exosome suspension was mixed with the stain solution and incubated for 4 min. The labeling reaction was stopped by adding an equal volume of 1% BSA. Labeled exosomes were ultra-centrifuged at 120,000 × g for 4 hrs, washed with PBS, and ultra-centrifuged again.

### Electron microscopy

Electron microscopy on purified exosomes was done as described [49]. Briefly, isolated exosomes were loaded onto carbon-coated grids, fixed in 2% paraformaldehyde, and washed in PBS. Resuspended pellets were deposited on Formvar-carbon coated EM grids and post-fixed in 2.5% glutaraldehyde, washed three times, contrasted with 2% uranyl acetate, and then examined with a JEOL 100CX transmission electron microscope (JEOL USA, Inc. Peabody, MA).

### Immunohistochemistry and light microscopy

Immunofluorescence analysis was done as we previously described [50, 51]. Cells were fixed in 100% methanol for 15 min at room temperature. For staining, samples were permeabilized for 15 min in freshly prepared PBS containing 0.25% Triton-X100, then blocked for 1h in 5% donkey serum, 0.1% fish gelatin, 0.2% Tween-20 and PBS. Samples were then incubated in 37°C water bath for 1 h with 1 mg/ml of primary antibody diluted in blocking solution. Samples were transferred to a 1:500 dilution of Goat anti-mouse IgG Rhodamine (Thermo Fisher Scientific, Waltham, MA) or Goat anti-rabbit IgG Fluorescein in blocking solution and incubated for in 37°C water bath for 1 h. Cells were mounted on a glass slide with mounting medium with DAPI (Vectashield, Burlingame, CA) and visualized with an inverted light microscope (Olympus IX81 and CellSens Dimension software, Center Valley, PA).

### Cloning of miRNA expression construct and transfection

DNA sequence for rno-miR-199a-3p and rno-miR-214-3p was retrieved from miRBase. Primer sequences flanking mature miRNA sequence was designed by using Primer-Blast (NCBI). Total genomic DNA was isolated from rBMSCs and PCR was done to amplify respective miRNA sequence. The resulting PCR product was cloned into pLKO.1 vector (Addgene, Watertown, MA) and recombinant clones were selected by restriction digestion and further by DNA sequencing analysis. Rat BMSCs were cultured on a 60 mm plate and transfected with purified miRNA expressing construct using either PE-mediated transfection or using *Trans*IT transfection kit (Mirus, Madison, WI). After 1- or 2-day post-transfection, transfected cells were used for further experiment.

## Results

### FAE model and isolation of exosomes from amniotic fluids

**Fig 1:**
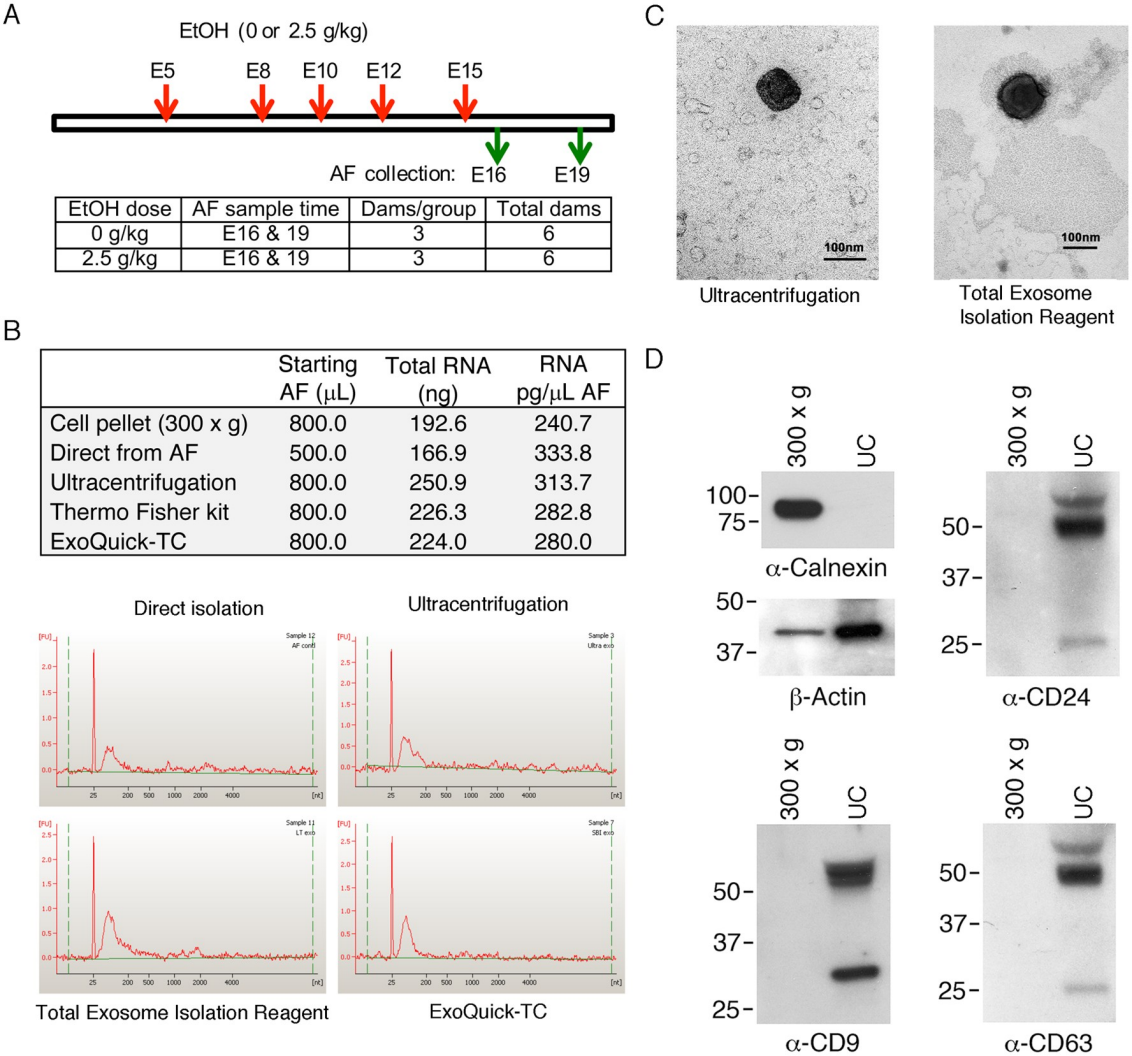
Isolation and characterization of amniotic fluid exosomes from rat fetal alcohol exposure (FAE) model. **(A)** Rat FAE model schematic. Timed pregnant rats were treated with water or 2.5 g/kg of 20% EtOH by oral gavage at E5, 8, 10, 12 and 15. Animals were euthanized at E16 or E19 for embryo collection (n = 3 each). **(B)** Comparison of amniotic exosome and exosomal RNA isolation by ultracentrifugation and two commercial kits (Total Exosome Isolation Reagent from Thermo Fisher and ExoQuick-TC from SBI). Cell pellet (300 x g) fraction and RNA isolation by direct lysis of AF are included for comparison. Graphs shown at the bottom are Bioanalyzer profiles of extracted exosomal RNA using a Nano kit. **(C)** Electron microscopic examination of amniotic exosomes purified by ultracentrifugation and Total Exosome Isolation Reagent (Thermo Fisher) (bar = 100 nm). **(D)** Immunochemical assessment of exosome purification by staining exosomal proteins with anti-Calnexin (endoplasmic marker), anti-Actin and exosomal markers (anti-CD9, CD24 and CD63). The level of the markers in the cell pellet (300 x g) fraction and ultracentrifugation fraction (UC) was compared.

To examine the effects of FAE on exosomal RNAs, groups of timed pregnant rats were treated with water or 20% EtOH in drinking water (2.5 g/kg by oral gavage) as described previously [34]. Amniotic fluids (AF) from embryos were collected on embryonic days 13, 16 and 19 (Fig 1A). There are numerous FAE models used to study the detrimental effects of EtOH on embryonic and postnatal development. Some involve generating EtOH-dependent rats and their water controls. These may then be bred with either similarly EtOH-dependent males or their water controls, with EtOH re-introduced daily for 4 hrs during pregnancy [52, 53]. This is an excellent model whose only drawback is the expense of maintaining a breeding colony of EtOH-dependent rats. Other models, including one we recently used to examine FAE consequences [34], involve EtOH administration to previously EtOH-naïve dams at various stages of pregnancy.

We found that AF volumes from E13 embryos were not sufficient for analysis. Therefore, we only used AFs from E16 and E19 embryos for the study. There are multiple ways of isolating exosomes, and a standard method has not been established for AF. Therefore, in pilot studies, we tested three different methods of exosome isolation: 1) differential centrifugation, 2) Total Exosome Isolation Reagent from cell culture media (Thermo Fisher Scientific, Waltham, MA), and 3) ExoQuick-TC (SBI, Palo Alto, CA). We found that the total RNA yields from three different methods were comparable, but the ultra-centrifugation method resulted in the highest yield (Fig 1B). We also compared the RNA profile on Bioanalyzer, with all three methods yielding similar results (Fig 1B). We have subsequently used the differential centrifugation method for our study. Example of AF volume harvest from our FAE model is shown in S2 Table in S1 File. Purification of exosomes were analyzed and compared by electron microscopy (Fig 1C) and confirmed by Western blotting analysis of exosomal marker (CD24, CD9, and CD63) expression. Endoplasmic calnexin and β-actin were used as controls (Fig 1D). Differences in exosome isolation between ultracentrifugation and ExoQuick-TC have been examined (S1 Fig in S1 File).

### RNA-seq analysis and verification

**Fig 2:**
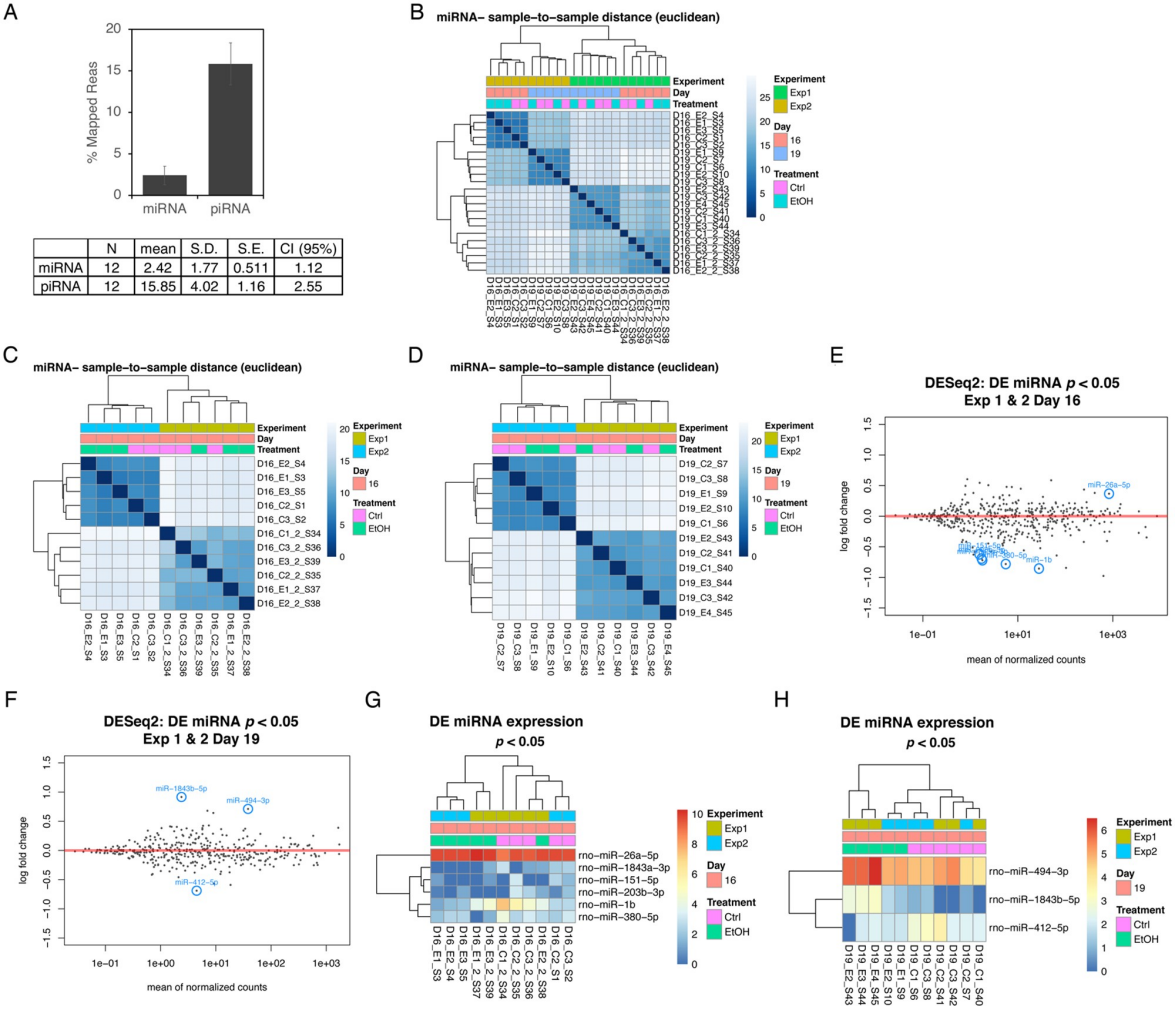
Bioinformatics analysis of miRNA-Seq data on amniotic exosomal miRNAs. **(A)** Processed RNA-seq data was mapped and found piRNA enriched in rat amniotic fluids compared to miRNA. Error bars represent 95% confidence interval. **(B)** Dendrogram was built to examine correlation among miRNAs in samples depending on the experiment (Exp1 and 2), collection time points (E16 and E19) and treatment (control vs. EtOH). Correlation among AF exosomal miRNAs for **(C)** E16 samples from Exp1 and 2 and **(D)** E19 samples from Exp1 and 2. (**E**) DESeq2 differential expression analysis for E16 samples from Exp1 and 2. **(F)** DESeq2 differential expression analysis for E19 samples from Exp1 and 2. **(G)** Selected six miRNAs with *p* < 0.05 differentially expressed in E16 samples from Exp1 and 2 are shown with circle. **(H)** Selected three miRNAs with *p* < 0.05 differentially expressed in E19 samples from Exp1 and 2 are shown with circle.

Total exosomal RNA was isolated by using miRNeasy kit (Qiagen) and subjected to the quality control assay. Bioanalyzer Pico Chip analysis (Agilent, Santa Clara, CA) showed the presence of abundant small RNAs (25–200 nt) in all AF samples (S2 Fig in S1 File). The library construction was done by using NEBNext Small RNA Library Prep Set for Illumina with spike-In reference RNA. Adapters and low-quality nucleotides were removed from raw fastq sequences. Adapter-free sequences were mapped to the Rat genome (rn5) using bowtie (v.1.1.2) using the command ‘-v 1 –k 100 –best–strata’ [43]. Average mapping rate was 40.92%, consisting of an average of 7.41% uniquely mapped reads and 92.59% and multi-mapped reads. Mapped reads were annotated to mature rat miRNA and piRNA. Rat miRNA annotations (rn5) were downloaded from miRBase [44]. Rat piRNA annotations (rn4) were downloaded from piRNABank [45]. Liftover of piRNA annotations rn4 to rn5 was performed since rn5 annotations were not available [46]. Bowtie alignments were annotated to miRNA and piRNA coordinates by requiring the alignment start site to be between -3nt to +2nt positions near the annotated start site and the alignment end site be between -2nt and +3nt of the annotated end site. Multi-mapped reads are distributed 1/n, where n is the number of mapping locations. Overall, rat amniotic datasets were significantly enriched with piRNA species compared to miRNA species (paired t-test; *p* = 3.12e-06) (Fig 2A).

DEseq2 [47] was used to determine differential expression of miRNA and piRNA species between control and EtOH treated groups for E16 and E19. Dendrogram was built to examine correlation among miRNAs in samples depending on experiment (Exp1 and 2), collection time points (E16 and E19) and treatment (control vs. EtOH). We found miRNA expression was correlated with experiment, but was not significantly correlated with treatment (Fig 2B). We also performed analysis separately for all AF miRNAs in E16 and E19 samples, but the expression of miRNAs was significantly correlated with experiment, but not with treatment (Fig 2C and 2D). DESeq2 differential expression analysis identified differentially expressed miRNAs in E16 samples and in E19 samples (Fig 2E and 2F). Selected number of miRNAs are shown with circle. We then focused on miRNAs showing significant changes upon EtOH treatment (*p* < 0.05). We found the selected 6 miRNAs in E16 samples and 3 miRNAs in E19 samples differentially expressed with *p* < 0.05 showed significant correlation with treatment (Fig 2G and 2H). This result shows significant experiment to experiment variations and batch effects in FAE-induced AF miRNA alterations in RNA-Seq analysis. However, we were able to identify a subset of miRNAs among them when we considered only miRNAs significantly affected by FAE (*p* < 0.05).

**Fig 3:**
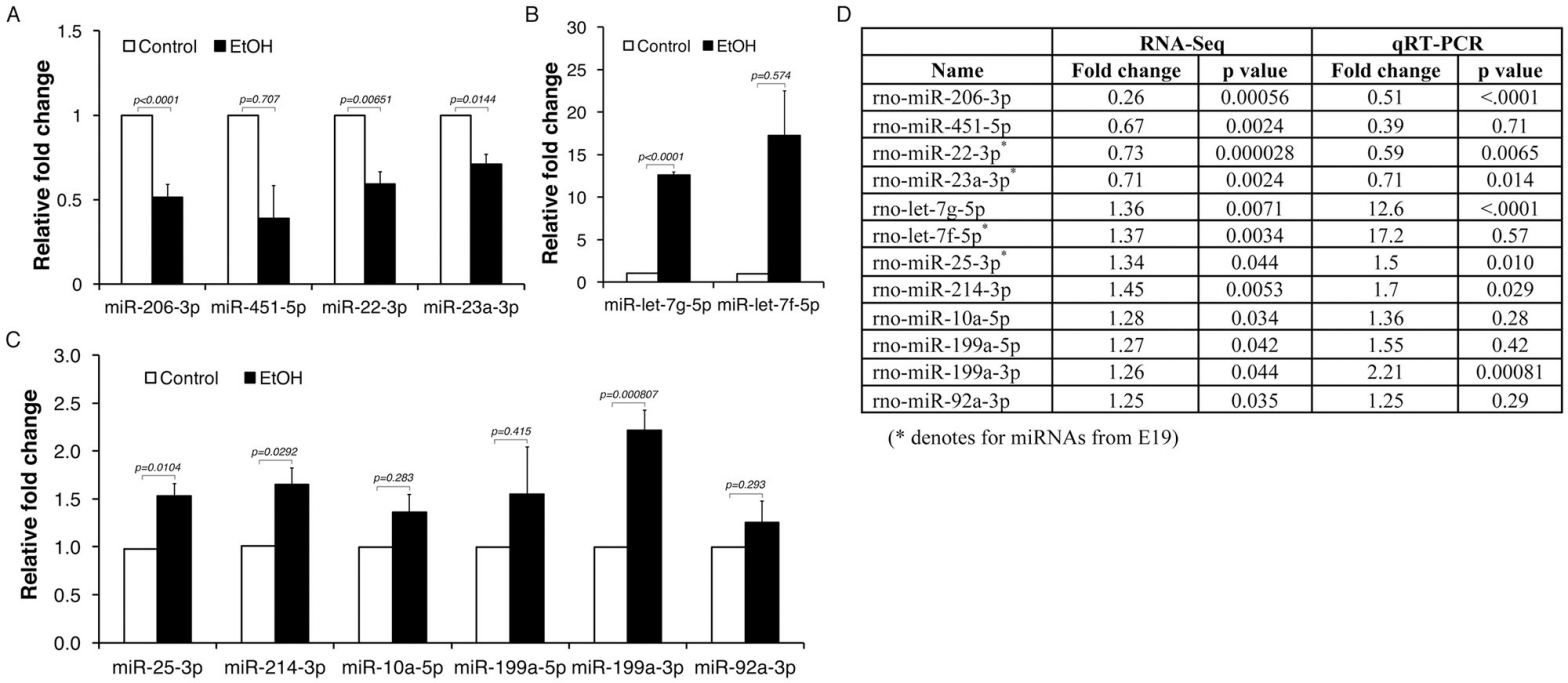
Verification of amniotic exosomal miRNA targets affected by FAE. Differential expression of amniotic exosomal miRNAs identified in FAE by RNA-Seq analysis were verified by qRT-PCR analysis for **(A)** downregulated in FAE and **(B** and **C)** upregulated in FAE. Summary of comparison between RNA-Seq and qRT-PCR analysis is shown in **(D)**. The *p* value was determined by one-way ANOVA and the error bars represent the standard error in triplicate samples.

To verify the FAE-induced changes in expression of AF exosomal miRNAs identified from RNA-Seq analysis, we selected 12 miRNA candidates based on *p* values and fold changes and performed quantitative RT-PCR analysis on total RNA isolated from AF exosomes (Fig 3A–3C).

Overall, we observed statistically significant FAE-induced alterations of AF miRNAs (*p* < 0.05) in seven out of twelve miRNA candidates tested. Fig 3D contains the summary results of the small RNA species found to be differentially expressed (*p* < 0.05) by RNA-Seq analysis and qRT-PCR verification. These data demonstrate maternal EtOH consumption in our animal model of FAE induced significant alterations in AF exosomal miRNA signatures that are considered to be fetal-origin.

**Fig 4:**
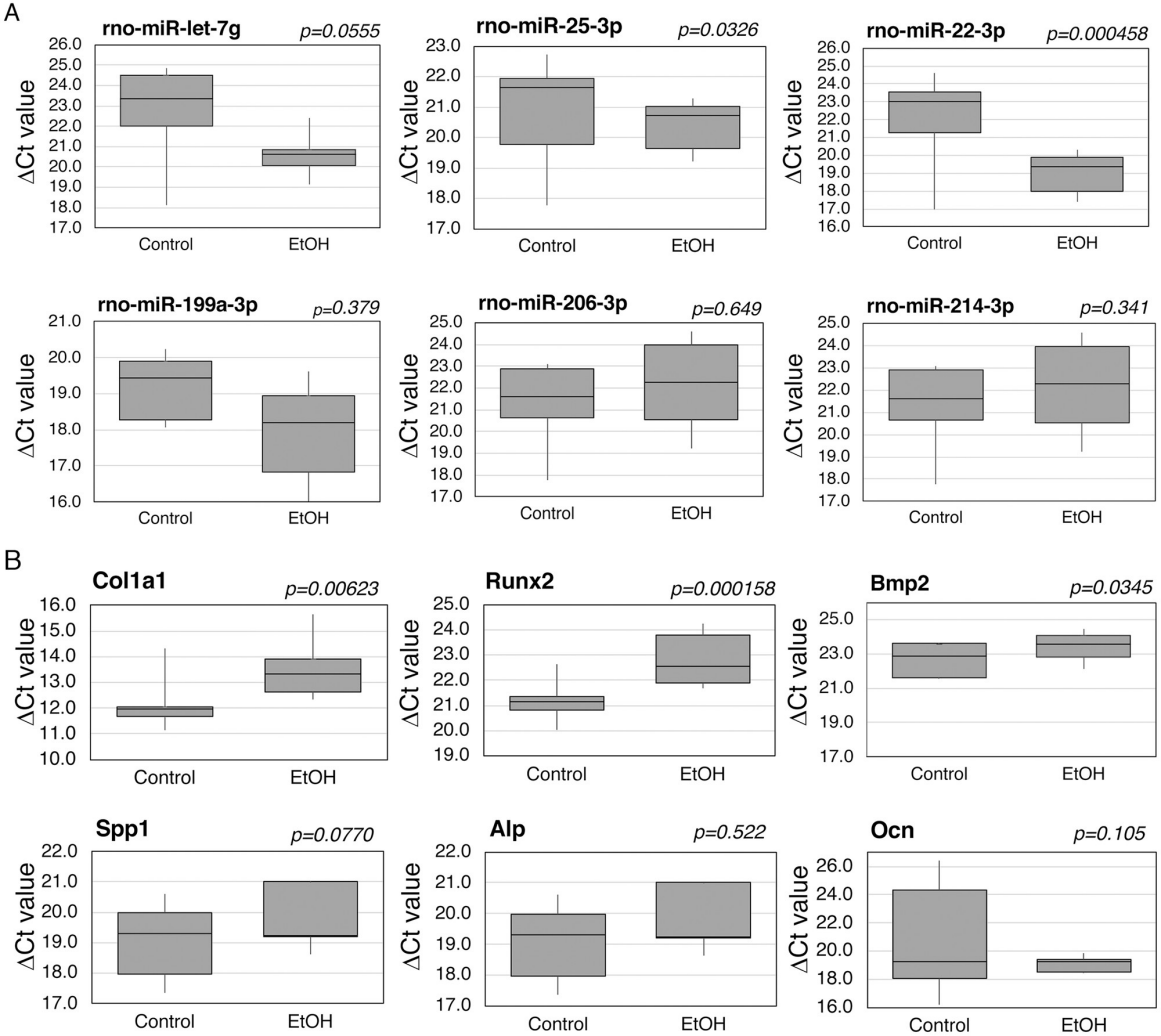
Effects of FAE on miRNAs and osteomarker expression in fetal embryo tissues. Whole rat fetal embryos at E16 from FAE model were used for total RNA isolation and **(A)** the levels of selected miRNAs and **(B)** osteogenic markers were determined by qRT-PCR analysis. The results of ΔCt values were compared between control group (n = 5) and EtOH group (n = 5). The *p* value was determined by one-way ANOVA and the error bar represents the standard error.

In addition, we have examined if the alterations in AF exosomal miRNAs induced by FAE can be detected in developing fetal embryos. Instead of focusing on a specific tissue of the embryo, we prepared total RNA from each embryo harvested at E16 and E19 (five embryos from one control dam and five embryos from one FAE dam) where we collected AF samples. We have determined and compared the levels of six selected miRNAs (rno-let-7g, 25-3p, 22-3p, 199a, 206-3p and 214-3p) (Fig 4A).

The result showed significantly (*p* < 0.05) increased level of rno-let-7g, rno-25-3p, rno-22-3p in embryo tissues from FAE group compared to control group while we found upregulated miRNAs (let-7g, 25-3p, 199a-3p and 214-3p) and downregulated miRNAs (206-3p and 22-3p) in FAE AF as shown in Fig 3. We have further determined effects of FAE on osteogenic marker levels in developing embryos (Fig 4B) and found several osteogenic genes (Col1a1, Runx2 and Bmp2) show significant downregulation in FAE group compared to control group (*p* < 0.05).

### Effects of AF exosomes from FAE on osteogenic potency of rat bone marrow stem cells

**Fig 5:**
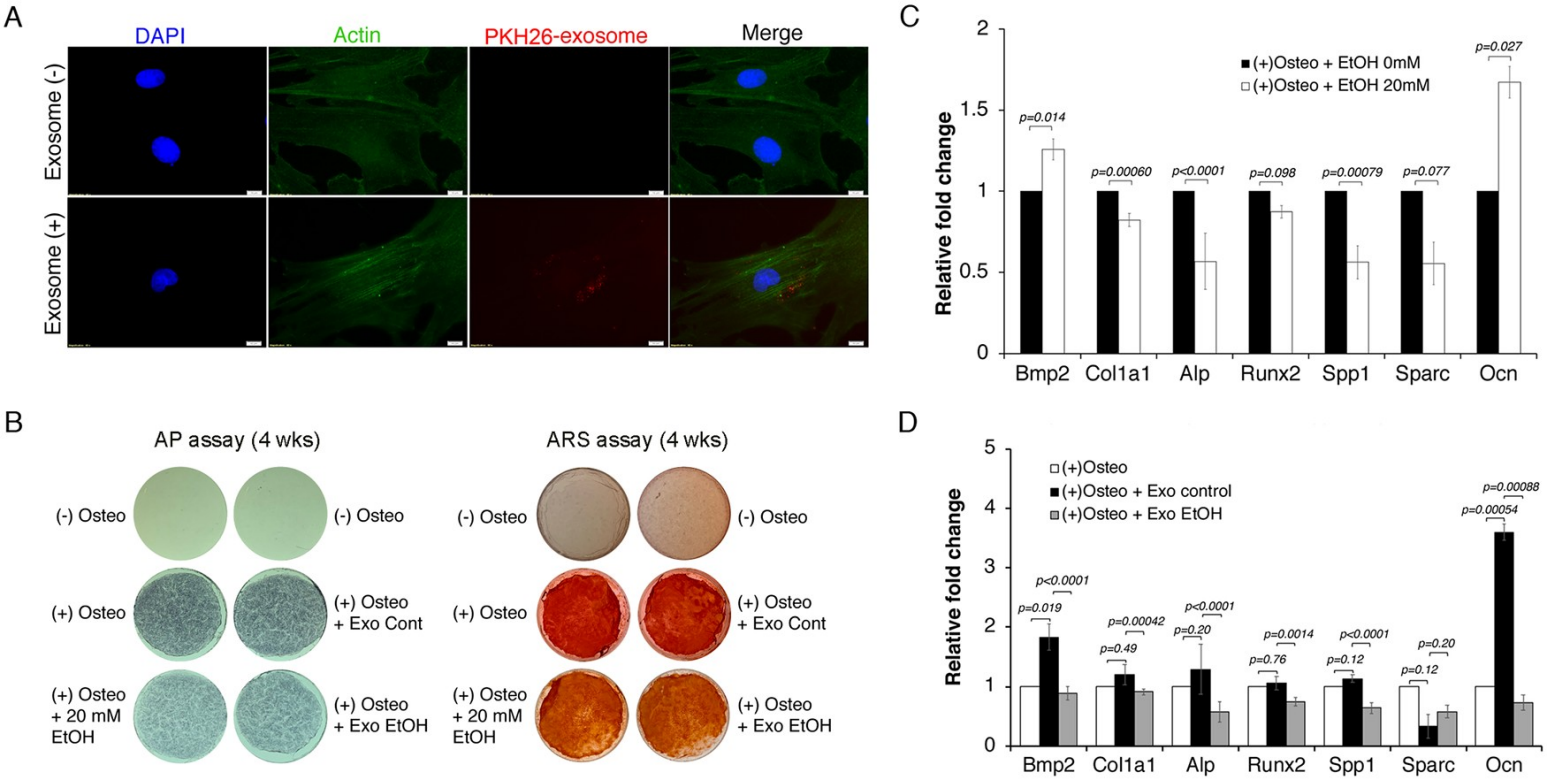
Uptake and functional effects of FAE amniotic exosomes on osteogenic potency of rat bone marrow stem cells. **(A)** After a 24 hr incubation period, PKH26-labeled exosomes were detected in the cytoplasm of β-Actin- and DAPI-labeled rBMSCs. Scale bar = 10 μm. Exosomes from amniotic fluids (control and FAE group) were purified, labeled with PKH26, and incubated with rBMSCs. After 24 hr incubation, cells were extensively washed and stained with anti-β-Actin antibody for cytoskeleton and DAPI for nucleus. Images were obtained with Olympus IX81 fluorescence microscope at 60× magnification. **(B)** Cells were induced for osteogenic differentiation in the absence or presence of 20 mM EtOH (alternating treatment by two-day treatment and two-day withdrawal) or exosome treatment every third day. After 4 weeks of osteogenic induction with 20 mM EtOH, exosome from control (+Exo Cont) or exosome from FAE (+Exo EtOH) group, cells were stained for alkaline phosphatase or mineral deposit by Alizarin Red staining. **(C)** Effects of EtOH (0 or 20 mM) 24-hr treatment on osteogenic potency of rBMSCs as determined by qRT-PCR analysis. **(D)** The effects of exosomes from control group (Exo control) or FAE group (Exo EtOH) on 24-hr osteogenic induction were determined. Relative fold changes in osteogenic markers from (+)Osteo-induction only, (+) Osteo-induction + Exo control and (+) Osteo-induction + Exo EtOH are shown. The *p* value was determined by one-way ANOVA and the error bar represents the standard error in triplicate samples.

We have recently reported EtOH’s significant effects on genetic and epigenetic signatures in human ESCs, hESC-derived neural stem cells and adult stem cells [6, 8, 35], and others showed that EtOH altered the differentiation potential of AF-derived stem cells [9]. EtOH readily crosses the placenta; consequently, peak fetal blood EtOH levels are similar to the mother [54]. Although EtOH clearance is increased in pregnancy [55], EtOH elimination capacity of the fetus is low, particularly in the early stages of pregnancy [56], and EtOH remains trapped in the AF leading to reabsorption by the fetus, thereby prolonging exposure time [55, 57]. Furthermore, there is a constant ingestion and secretion of bioactive amniotic exosomes and exRNA in AF by the fetus. We hypothesized that exRNAs present in AF may have effects on stem cells and thus, FAE may have significant effects on exRNA-mediated stem cell regulation. To test our hypothesis, we tentatively examined the uptake of purified AF exosomes by labeling them with PKH26 and incubated the labeled AF exosomes with rat bone marrow stem cells (rBMSCs). Labeled AF exosomes were taken up by rBMSCs and localized mostly in the cytoplasm (Fig 5A).

To evaluate functional effects of FAE AF exosomes on osteogenic differentiation, we incubated rBMSCs with purified AF exosomes from control and FAE group under osteogenic induction for 4 weeks. Its effect on osteogenic differentiation was compared with cells treated with EtOH (20 mM) by alkaline phosphatase staining and also the level of mineral deposit by alizarin red staining (Fig 5B). The result showed noticeable reduction in AP activity and ARS staining in cells treated with EtOH and similarly in cells treated with exosomes from FAE group compared to the control group.

Next, to evaluate possible functional effects of FAE AF exosomes on stem cell regulation we first incubated rBMSCs with EtOH (20 mM) for 24 hrs to compare with the effects of purified FAE AF exosome incubation on osteogenic differentiation of rBMSCs. EtOH treatment of rBMSCs for 24 hrs resulted in reduced activation of some molecular markers, significantly Col1a1, Spp1 and Alp, during osteogenic differentiation (Fig 5C). We next tested if FAE AF exosomes could recapitulate the suppressive effect of EtOH on osteogenic differentiation of rBMSCs by incubating them with purified AF exosomes from either control (*Exo control*) or EtOH-exposed (*Exo EtOH*) animals. When exosomes from control group (*Exo control*) were added to rBMSCs under osteogenic differentiation, we found significant induction of Bmp2 and Ocn (Fig 5D). However, when AF exosomes from FAE group (*Exo EtOH*) were added to rBMSCs undergoing osteogenic differentiation, we observed significant suppression of most osteogenic markers compared to controls (*Exo control*) (Fig 5D). These data demonstrate that molecular effects of FAE can be transferred into neighboring microenvironment *via* exosomal delivery. We have also examined the level of osteopontin, one of osteogenic marker protein, by Western analysis. The result showed the reduction of osteopontin protein with EtOH treatment and also after treatment with AF exosomes from EtOH-exposed (Exo EtOH) animals compared to treatment with AF exosomes from control group (Exo Cont) (S4 Fig in S1 File).

### Molecular function of AF miRNAs affected by FAE in osteogenic differentiation of rBMSCs

**Fig 6:**
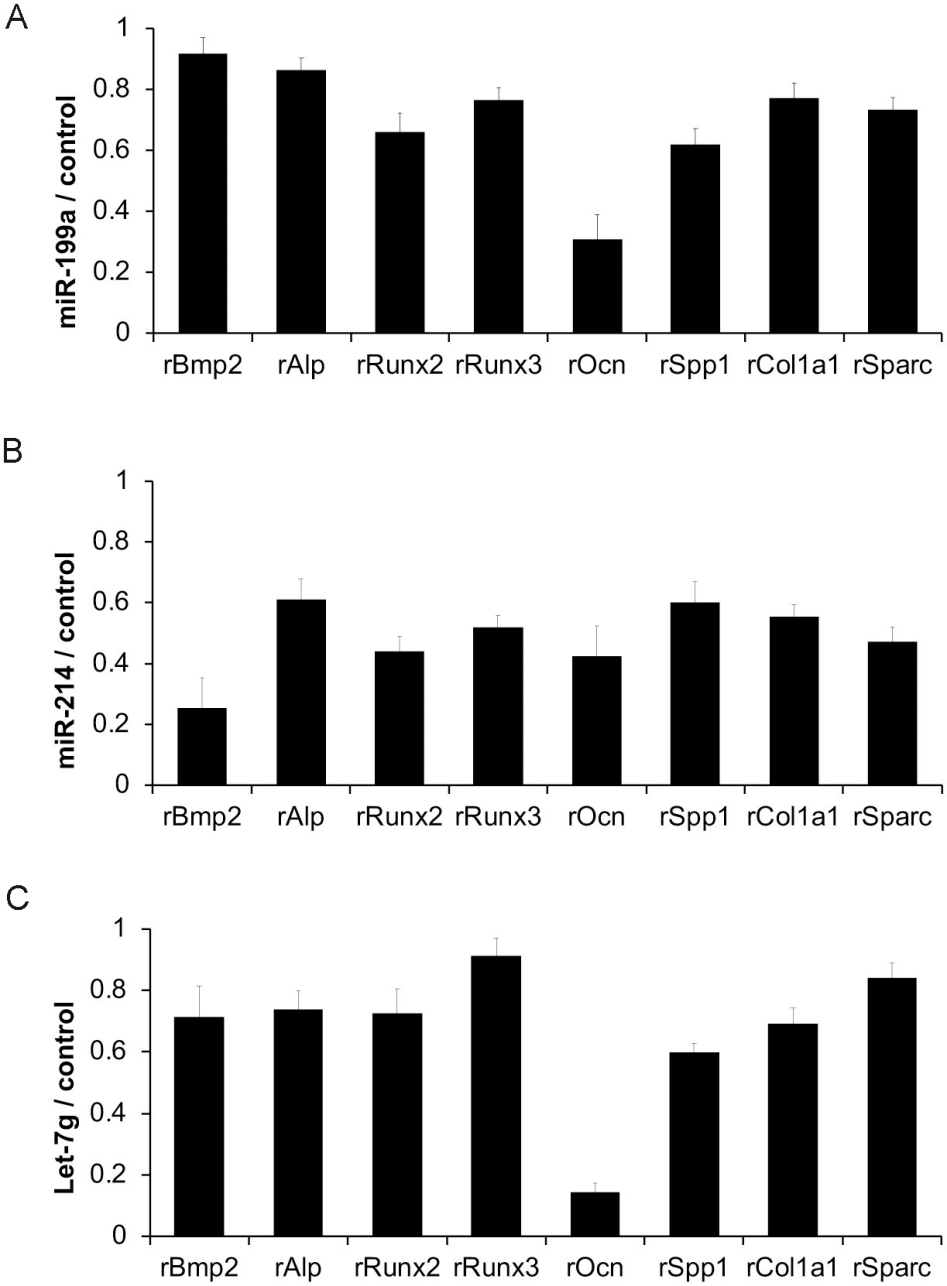
Effects of miRNAs upregulated in rat AF exosome by FAE on osteogenic differentiation of rat bone marrow stem cells. Molecular effects of miR-199a-3p, miR-214-3p and let-7g on osteogenic differentiation of rBMSCs were determined by transfection study to undifferentiated rBMSCs. Effects of **(A)** miR-199a-3p, **(B)** miR-214 and **(C)** let-7g on osteogenic markers in the presence of osteo-induction (for 24 hrs) were examined. The *p* value (<0.05) was determined by one-way ANOVA and the error bar represents the standard error in triplicate samples.

We demonstrated that FAE alters AF exosomal miRNAs (Figs 2 and 3) and separately showed that FAE exosomal transfer to rBMSCs had significant suppressive effects on osteogenic potency (Fig 5). Therefore, we next tested if AF exosomal miRNAs altered by FAE have a direct role in osteogenic differentiation of rBMSCs. Thus, we cloned three miRNA candidates (199a-3p, 214-3p and let-7g) that were significantly upregulated in AF exosomes by FAE. Undifferentiated rat BMSCs were transfected with a vector control- or miRNA-expressing construct and we initially examined if overexpression of miRNA candidates had any effect on osteogenic marker expression during differentiation of rBMSCs (Fig 6).

Overexpression of miR-199a-3p, miR-214 and let-7g in rBMSCs resulted in decreased levels of osteogenic differentiation markers compared to the control (Fig 6). This result demonstrated that the altered levels of these miRNAs due to cellular challenges such as EtOH exposure could affect osteogenic differentiation in rBMSCs.

In summary, our findings demonstrate that (1) FAE induces alterations of miRNAs such as miR-199-3p, miR-214-3p and let-7g, (2) these miRNAs are packaged in exosomes as extracellular RNA and presented in amniotic fluids of embryos, and (3) these miRNAs have functional roles in stem cell regulation, maintenance and differentiation.

## Discussion

Alcohol abuse during pregnancy is known to cause abnormalities in developing embryos and lead to fetal alcohol spectrum disorders (FASDs) [2]. However, the specific mechanisms by which alcohol mediates these injuries have yet to be determined [3, 4]. During embryogenesis there are significant intercellular communications between the mother and the developing embryo. Amniotic fluid (AF) serves as an important medium in the trafficking of molecular information between the mother and the fetus. It was also shown that amniotic fluid contains secreted exosomes with extracellular RNA (exRNA) from both maternal and developing fetal tissues [28, 29, 32]. Throughout the development, the AF is in direct contact with the fetus. RNAs in the AF can be absorbed through non-keratinized fetal skin and gastrointestinal tract, and thus, affect stem cell differentiation in the fetus. [58, 59]. Furthermore, alcohol was shown to significantly affect exosomal protein and RNA content in adult tissues [23, 24]. Thus, maternal alcohol consumption can significantly affect the microenvironment surrounding the developing embryo, including the biogenesis and the content of exosomal exRNA. In this study, we have tested our hypothesis that maternal alcohol consumption during pregnancy in the animal model of fetal alcohol exposure alters the biogenesis of amniotic exosomal RNA that may have biofunction in stem cell development.

From RNA-Seq analysis, we have identified AF exosomal miRNAs that are potentially affected by FAE. We have further verified the FAE-induced changes in the level of selected AF exosomal miRNAs. The importance of epigenetic regulation including noncoding RNAs such as miRNAs has been demonstrated in alcohol-induced pathogenesis. Studies have reported that EtOH alters a variety of miRNAs that can control alcohol-induced pathologies or dysfunctions in multiple organs. Furthermore, alterations of miRNA expression patterns in response to developmental alcohol exposure have been reported. It has been suggested that miRNAs are vulnerable to alcohol exposure and may contribute to the detrimental effects on fetal development [60–62]. Potential fetal tissue origin of these AF miRNAs altered by FAE was demonstrated by the coherent changes in miRNA level in total fetal embryo tissue (Fig 4A).

The results of our study can provide scientific underpinning for the known phenotypes associated with FASD. For instance, prenatal EtOH exposure can cause short stature, decrease in length of bones, lower level of skeletal maturity, and delay ossification [63], which can be explained with the miRNA alterations that we found in the AF. MiRNAs 25-3p and 214-3p both interfere with the BMP/SMAD signaling, which indirectly activates regulators of osteoblast differentiation such as RUNX2 [64]. MiRNA 25-3p binds to the 3’ Untranslated Region (UTR) of SMAD5 mRNA, a regulator of the BMP/SMAD signaling, and silences it [65]. Similarly, miRNA 214-3p binds to the 3’ UTR of BMP2, inhibiting its expression [66]. Both of these miRNAs are regulated with long non-coding RNAs that are normally expressed in increasing amounts during osteogenic differentiation [65]. Therefore, up-regulation of these miRNAs observed in our results (Figs 3D and 4A) can inhibit osteogenic differentiation. In contrast, EV-encapsulated miRNA-22-3p from BMSCs have been shown to promote osteogenic differentiation in neighboring BMSCs and increase matrix mineralization [67]. Hence, the up-regulation of miRNAs 25-3p and 214-3p as well as down-regulation of miRNA 22-3p all can lead to inhibition of osteogenic gene expression observed in Fig 5, prevent osteogenic differentiation, and cause the osteogenic deficits observed in FAE.

Moreover, to further confirm this effect of alcohol on bone metabolism, we analyzed the effect of FAE amniotic exosomes on expression of osteogenic markers. rBMSCs exposed to exosomes derived from FAE AF exhibited a significant decrease in osteogenic potency compared to the control group. This indicates that alcohol affects osteogenic differentiation through altering exosomes, making miRNAs a very likely candidate for such effect. Previous studies have reported an association between elevated miRNA 22-3p levels and increased expression of RUNX2 and OCN [67]. Therefore, a decrease in miRNA 22-3p can lead to a decrease in these osteogenic markers, which was also observed in our experiments (Fig 5D). Furthermore, the results of this experiment are consistent with previous studies that found a decrease in collagen type 1 gene expression and alkaline phosphatase activity following presence of alcohol on human mesenchymal bone marrow stem cells [68].

Defining the effect of maternal alcohol consumption on amniotic exRNA content may be used to develop clinical tools to monitor the effects of alcohol on the pregnancy progress and fetal development. More importantly the identification of exRNA species associated with FASDs will be beneficial towards development of biomarkers for early detection and interventions to prevent or mitigate EtOH’s teratogenic effects. Similar to previous studies [26, 27, 69, 70], our study supports the use of miRNAs as a non-invasive tool to diagnose teratogenic effects of alcohol on the fetus. FASD is diagnosed through characteristic facial dysmorphology, growth restriction, and neurodevelopmental abnormalities [71]. However, many of the prenatally exposed infants do not necessarily exhibit these physical features early in life, making diagnosis of FAE harder [69]. Moreover, early diagnosis of FASD and other alcohol-related fetal disorders is important to prevent further damage and alleviate the defects that arise later in the life. Contrary to the previous studies which researched effects of alcohol on miRNAs in maternal plasma or circulation [26, 27, 69], we studied such effect in the AF and found a change in a different set of miRNAs. This difference in the miRNA profile of the previous studies and this study is expected, as miRNAs are not just released from necrotic or injured cells but also actively secreted and therefore, different body fluids have different expression profiles of miRNAs [72]. One of the disadvantages of using the miRNAs in the circulation is the fact that their origin is rather unclear [73]. Alcohol causes injury or inflammation in multiple organs of the body including the liver, brain, heart, and pancreas [73]. Moreover, blood cells are a major source for circulating miRNAs [74]. Therefore, increases in circulatory miRNAs may be due to injury or necrosis of a secondary maternal tissue that does not necessarily affect development, which decreases the specificity and accuracy of those miRNAs as a biomarker [73]. In contrast, the AF represents a pure fetal sample of the nucleic acids [32]. The miRNA alterations found in this study can also be used in developing treatments for FASD.

Further *in vivo* analysis will be required to confirm the effects of exosomal transfer of EtOH’s effects on stem cell potency *in utero*. One meritorious future study will be to identify potential molecular targets of AF exosomal miRNAs that are important in mediating the effects of FAE in stem maintenance and differentiation. Future studies can speculate whether returning miRNA activity in the AF to the normal levels using miRNA mimics and inhibitors can mitigate or prevent the negative effects of alcohol on development. Similar uses of miRNA mimics and inhibitors have also been suggested in stem cell-based therapy in adults [70].

## Supporting information

S1 File (DOCX)
