# Supplementary material for: Molecular consequences of fetal alcohol exposure on amniotic exosomal miRNAs with functional implications for stem cell potency and differentiation
Source: PLoS One. 2020 Nov 16;15(11):e0242276. doi: 10.1371/journal.pone.0242276 (PMC7668603; doi:10.1371/journal.pone.0242276)
Supplement: S1 File — (DOCX) [file pone.0242276.s001.docx]

**SUPPORTING Information**

**S1 Table. miRNA sequences used for qRT-PCR verification**

| **miRNA** | **Sequence** |
| --- | --- |
| rno-miR-451-5p | AAACCGTTACCATTACTGAGTT |
| rno-miR-214-3p | ACAGCAGGCACAGACAGGCAG |
| rno-let-7g-5p | TGAGGTAGTAGTTTGTACAGTT |
| rno-miR-10a-5p | TACCCTGTAGATCCGAATTTGTG |
| rno-miR-92a-3p | TATTGCACTTGTCCCGGCCTG |
| rno-miR-199a-5p | CCCAGTGTTCAGACTACCTGTTC |
| rno-miR-199a-3p | ACAGTAGTCTGCACATTGGTTA |
| rno-miR-22-3p | AAGCTGCCAGTTGAAGAACTGT |
| rno-miR-23a-3p | ATCACATTGCCAGGGATTTCC |
| rno-let-7f-5p | TGAGGTAGTAGATTGTATAGTT |
| rno-miR-206-3p | TGGAATGTAAGGAAGTGTGTGG |
| rno-miR-25-3p | CATTGCACTTGTCTCGGTCTGA |
| RNU1A | CGACTGCATAATTTGTGGTAGTGG |

**S2 Table. Isolation of amniotic fluid from rat FAE model**

| **E16** | **Total AF (aprx. ml)** |
| --- | --- |
| **EtOH group** |  |
| Rat 1 (embryo #1 - 12) | 3.5 |
| Rat 2 (embryo #13 – 22) | 2.5 |
| Rat 3 (embryo #23 - 32) | 3.0 |
|  |  |
| **Control group** |  |
| Rat 1 (embryo #1 - 12) | 3.0 |
| Rat 2 (embryo #13 - 26) | 3.4 |
| Rat 3 (embryo #27 - 39) | 3.7 |
|  |  |
| **E19** | **Total AF (aprx. ml)** |
| **EtOH group** |  |
| Rat 1 (embryo #1 - 12) | 5.0 |
| Rat 2 (embryo #13 - 24) | 5.0 |
| Rat 3 (embryo #25 - 35) | 4.5 |
|  |  |
| **Control group** |  |
| Rat 1 (embryo #1 - 13) | 5.3 |
| Rat 2 (embryo #14 - 27) | 5.0 |
| Rat 3 (embryo #28 - 38) | 5.0 |

**
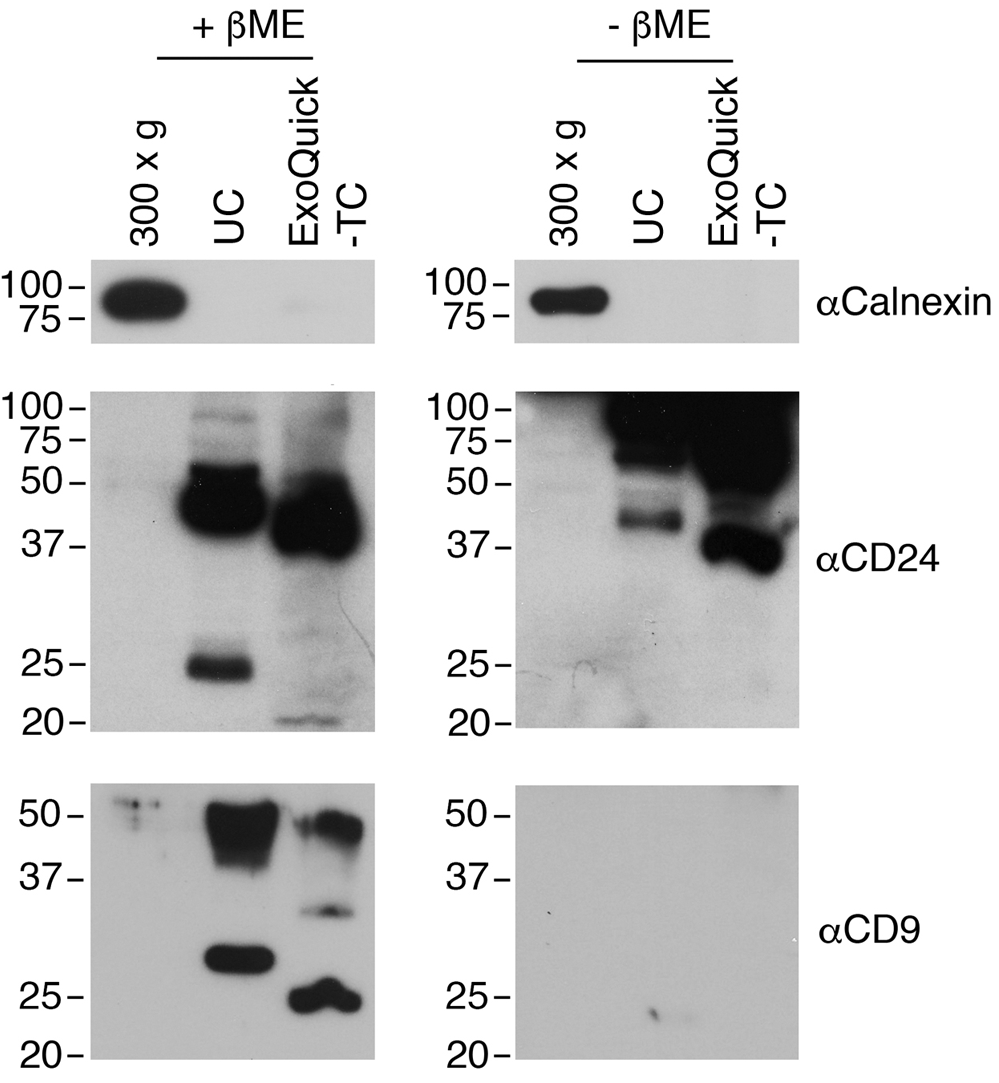
**

**S1 Figure. Characterization of amniotic exosomes purified from rat FAE model.** Amniotic exosomes were purified by using ultracentrifugation (UC) and ExoQuick-TC kit (SBI) and analyzed by Western blotting for endoplasmic marker (Calnexin) or two exosome markers (CD24 and CD9). Samples were prepared without or with reducing agent, β-mercaptoethanol (βME). A cell pellet fraction from 300 x g spin was included as a control.

**
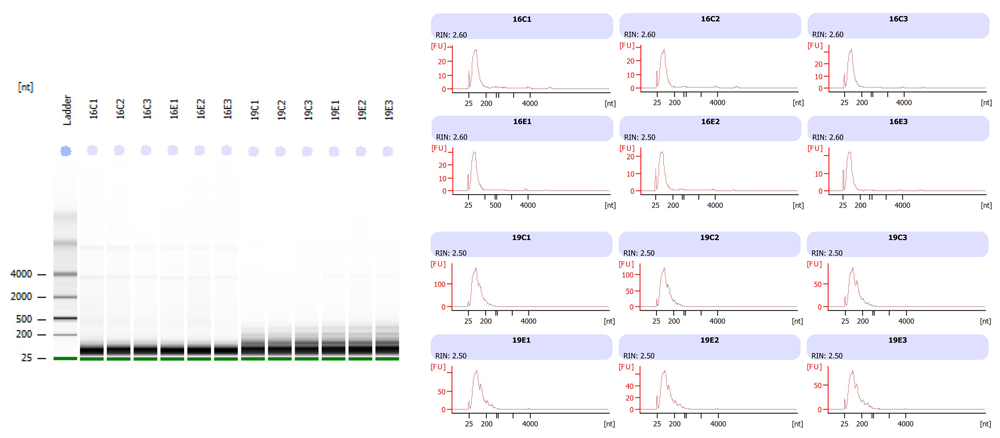
**

**S2 Figure. Characterization of exosomal RNA used for RNA-Seq analysis.** Total small RNA purified from AF exosomes (C1-C3: three biological replicates of control group and E1-E3: three biological replicates of EtOH group from E16 or E19) were analyzed by Bioanalyzer Pico kit.

**S3 Figure. Full-length western blot images used for Fig.1 and Fig. S1.**


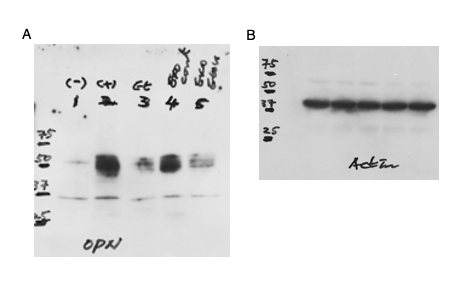


**S4 Figure. Western analysis for osteogenic marker.** Cells were induced for osteogenic differentiation in the absence or presence of 20 mM EtOH (alternating treatment by one-day treatment and one-day withdrawal) or exosome treatment every two days. After 7 days of osteogenic induction with 20 mM EtOH, exosome from control (+Exo Cont) or exosome from FAE (+Exo EtOH) group, cells were examined for the expression of osteopontin (SPP1) (1:1,000, Santa Cruz Biotechnology, sc-21742), one of osteogenic markers. Equal sample loading was confirmed by staining with anti-Actin antibody (1:3,000, Sigma).
